# *Vibrio* diversity and dynamics in the Monterey Bay upwelling region

**DOI:** 10.3389/fmicb.2014.00048

**Published:** 2014-02-12

**Authors:** Sarah Mansergh, Jonathan P. Zehr

**Affiliations:** Ocean Sciences Department, University of California at Santa CruzSanta Cruz, CA, USA

**Keywords:** *Vibrio*, upwelling, Monterey Bay, seasonal variability, 16S rRNA gene diversity

## Abstract

The *Vibrionaceae* (*Vibrio*) are a ubiquitous group of metabolically flexible marine bacteria that play important roles in biogeochemical cycling in the ocean. Despite this versatility, little is known about *Vibrio* diversity and abundances in upwelling regions. The seasonal dynamics of *Vibrio* populations was examined by analysis of 16S rRNA genes in Monterey Bay (MB), California from April 2006–April 2008 at two long term monitoring stations, C1 and M2. *Vibrio* phylotypes within MB were diverse, with subpopulations clustering with several different cultured representatives including *Allivibrio* spp., *Vibrio penaecida*, and *Vibrio splendidus* as well as with many unidentified marine environmental bacterial 16S rRNA gene sequences. Total *Vibrio* population abundances, as well as abundances of a *Vibrio* sp. subpopulation (MBAY Vib7) and an *Allivibrio* sp. subpopulation (MBAY Vib4) were examined in the context of environmental parameters from mooring station and CTD cast data. Total *Vibrio* populations showed some seasonal variability but greater variability was observed within the two subpopulations. MBAY Vib4 was negatively associated with MB upwelling indices and positively correlated with oceanic season conditions, when upwelling winds relax and warmer surface waters are present in MB. MBAY Vib7 was also negatively associated with upwelling indices and represented a deeper *Vibrio* sp. population. Correlation patterns suggest that larger oceanographic conditions affect the dynamics of the populations in MB, rather than specific environmental factors. This study is the first to target and describe the diversity and dynamics of these natural populations in MB and demonstrates that these populations shift seasonally within the region.

## Introduction

The *Vibrionaceae* (*Vibrio*) are a group of physiologically-flexible marine bacteria that are ubiquitous in ocean waters and have been identified in most marine ecosystems (Wietz et al., 2010). *Vibrio* have the distinctive ability to break down and utilize many carbon, nitrogen, and phosphorus substrates (McDougald and Kjelleberg, 2006; Thompson and Polz, 2006; Dryselius et al., 2008; Lai et al., 2009; Salter et al., 2009) and their production of the external enzymes chitinase and laminarinase provide access to abundant nutrients that are unavailable to other organisms (Svitil et al., 1997; Riemann et al., 2000; Ansede et al., 2001; Alderkamp et al., 2007; Murray et al., 2007). In addition to their diverse metabolic capabilities, *Vibrio* species have developed adaptive responses to starvation and environmental stress which include conversion to an ultramicrobacterial morphology (<0.4 μm diameter) (Denner et al., 2002) and retention of a high concentration of rRNA as a “stimulation ready” response that may contribute to this group's rapid growth potential after nutrient influxes (Eilers et al., 2000). Despite these capabilities, there have been few studies in oceanic upwelling regions, where metabolic flexibility, rapid nutrient response, and highly developed stress protection mechanisms should prove to be ecologically advantageous during changing environmental regimes.

Monterey Bay (MB) is an open embayment along the California coast that is distinguished by a near-shore deep-water canyon where periodic upwelling events sustain diverse sea life. Circulation within MB is variable and influenced by recently upwelled waters as well as offshore waters from the California and Davidson Currents that enter MB during relaxation events (Rosenfeld et al., 1994). Three hydrographic seasons have been defined within MB—an upwelling or “cold water phase” that usually occurs from mid-February through August, an oceanic period or “warm water phase” from mid-August through mid-October and a Davidson Current or “low thermal gradient” phase between mid-November and mid-February (Skogsberg and Phelps, 1946; Breaker and Broenkow, 1994). The periodic influx of nutrient-enriched waters into MB via upwelling results in a highly productive ecosystem presenting a unique environment for the study of *Vibrio* population dynamics.

*Vibrio* species are common isolates from MB waters but most studies have concentrated on pathogenic representatives (Kenyon et al., 1984; Kaysner et al., 1987; Miller et al., 2006). One phylogenetic screening study found that *Vibrio*-specific sequences made up 3.1% of the genetic information within a FOSMID library developed from a 100 m deep sample (Suzuki et al., 2004). *Vibrio* populations are estimated to average only about 1% of marine bacterial populations worldwide (Thompson and Polz, 2006) but it has also been suggested that differences in vertical distributions may be more significant than differences in geographic distribution for *Vibrio* populations, and thus, may make up a more significant portion of subsurface bacterial populations (Simidu and Tsukamoto, 1985).

**Figure 1:**
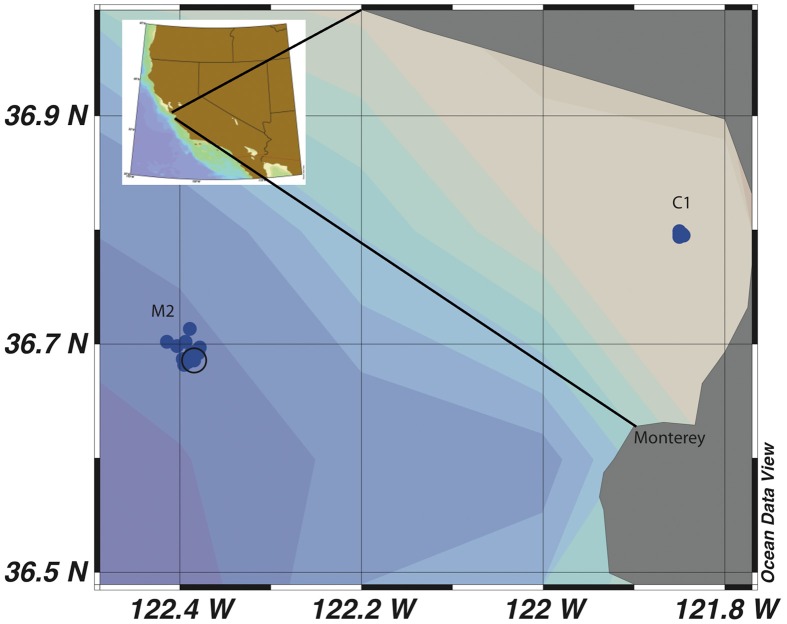
**Map of sample sites C1 and M2 in Monterey Bay, California**. Each individual blue dot represents a sampling event and the black circle is the identified coordinate of the mooring station at M2.

This study was designed to determine the seasonal variability of *Vibrio* populations in MB by examining total *Vibrio* population dynamics as well as two subpopulations, a *Vibrio* sp. subpopulation (MBAY Vib7) and an *Allivibrio* sp. subpopulation (MBAY Vib4), and to analyze how population diversity changes with upwelling, season, and environmental factors. MB has a system of long term monitoring stations (Pennington and Chavez, 2000) and two of the stations within this network, C1 and M2 (Figure 1), were chosen as sample sites for this study. C1 is a coastal site that is influenced by aged upwelled waters and summer relaxation events. M2 is an outer Bay site that is influenced by upwelled waters that mix with California Current and Davidson Current waters. The examination of *Vibrio* population dynamics at these two different locations can provide insight into the effect of upwelling characteristics on *Vibrio* abundances.

## Materials and methods

### Sample collection

Seawater samples were collected from MB at stations C1 (36.797 N; 121.847 W; Figure 1) and M2 (36.697 N; 122.378 W; Figure 1) during cruises of the MB Time Series project undertaken by the Biological Oceanography Group (BOG) at the Monterey Bay Aquarium Research Institute (MBARI). Samples were collected from April 2006 through April 2008 on a periodic basis spanning 19 sampling dates. Samples were collected using a SeaBird 911 CTD rosette equipped with physical sensors described by Pennington and Chavez (2000). A total of 82 samples were collected at station C1 at 5 m (15 samples), 10 m (17 samples), 20 m (18 samples), 30 m (17 samples), and 200 m (15 samples). At station M2 a total of 86 samples were collected at 5 m (12 samples), 10 m (14 samples), 20 m (14 samples), 40 m (16 samples), 100 m (16 samples), and 200 m (16 samples). For each sample 1–2 liters of seawater were filtered using gentle peristaltic pumping through sequential in-line 25 mm diameter 10 μm pore-size PE filters (GE Osmonics, Minnetonka, MN, USA) and 25 mm diameter 0.2 μm pore-size Supor 200 membrane filters (Pall Corporation, Port Washington, NY, USA). The filters were transferred to 1.5 mL polypropylene microcentrifuge tubes containing 0.2 g of 0.1 mm and 0.5 mm diameter autoclaved glass beads (BioSpec Products, Bartlesville, OK, USA). Samples were flash frozen in liquid nitrogen before transfer to a −80°C freezer onshore for storage until nucleic acids were extracted.

### Environmental data

Upwelling indices (UI) of both monthly and daily averaged upwelling conditions were obtained for 36°N 122°W from the Pacific Fisheries Environmental Laboratory (PFEL, Pacific Grove) for determination of upwelling patterns (http://www.pfeg.noaa.gov/products/PFEL/modeled/indices/PFELindices.html). Units are given as m^3^ s^−1^ 100 m of coastline-^1^ as the average amount of water upwelled through the bottom of the Ekman layer each second along each 100 m of coastline on a scale of about 200 miles. Surface data for nitrate, chlorophyll *a*, and temperature was obtained from the M2 mooring from MBARI LOBOviz (http://www.mbari.org/lobo/loboviz.htm). The BOG at MBARI provided environmental data from CTD measurements as well as surface (<10 m) phytoplankton concentrations, which were analyzed as described by Chavez et al. (1990). Specific phytoplankton groups (*Synechococcus*, total diatom, total dinoflagellate, and total phytoplankton) were chosen for analysis to assess the influence of different phytoplankton regimes on the variability of *Vibrio* groups in the water column.

### DNA extraction

DNA was extracted from the 0.2 μm pore-size filters using the Qiagen DNeasy Plant Kit (Hilden, Germany) as described by Moisander et al. (2008) with modifications to improve DNA recovery. Cells were lysed by a triplicate run of freeze-thaw steps in liquid nitrogen followed by a 65°C water bath. Cells were further disrupted by a 2-min agitation of bead beating (Mini bead beater 96, BioSpec Products, Bartlesville, OK, USA) and DNA yield was increased with the addition of 0.45 μ L of Proteinase K and incubation at 55°C for 1 h. AE buffer was used as the elution medium. Two duplicate elutions of 25 μ L each were combined for a final elution volume of 50 μ L. Extracts were stored at −20°C until use.

### Amplification, cloning and sequencing of *Vibrio*-specific 16S rRNA gene

Samples were selected from 2007 to 2008 from 5 to 200 m depths at both station C1 and M2 to construct clone libraries of amplified 16S rRNA gene sequences from the MB. A total of 65 samples were processed for inclusion in the libraries. The PCR amplification utilized a universal forward primer (27F, Table 1) and a *Vibrio* specific reverse primer (680R, Table 1) and followed the two-phase PCR amplification technique outlined by Thompson et al. (2004b). Samples were amplified using Invitrogen *Taq* polymerase (Carlsbad, California), on a BioRad thermal cycler (Hercules, California). The PCR products were gel purified and cloned into pGEM-T vectors (Promega, Madison WI) using manufacturer's guidelines. Ten to fifteen clones were chosen from each sample, for a total of 911 clones, and prepared for sequencing with the Montage Plasmid Miniprep kit (Millipore, Billerica, MA) according to manufacturer's protocols. Cloned inserts were sequenced at the UC Berkeley DNA Sequencing Facility using T7 primers and analyzed on an Applied Biosystems 3730xl DNA Analyzer. Sequences were aligned and compared to published sequences using the Ribosomal Database Project (RDP) on-line interface (Cole et al., 2007, 2009) and were quality checked for chimeras using the RDP Chimera check program and Bellerophon (Huber et al., 2004). Phylogenetic analysis of 827 sequences was conducted in ARB (Ludwig et al., 2004) and neighbor-joining phylogenetic trees were constructed using the Jukes-Cantor correction. The distance matrix derived from the neighbor joining analysis was used in DOTUR for assignment of operational taxonomic units (OTUs) (Schloss and Handelsman, 2005). All sequences were submitted to the National Center for Biotechnology Information (NCBI) GenBank database as accession numbers KF941543–KF942369.

**Table 1:** **Primers and probes for PCR and qPCR (written 5′−>3′)**.

**16S rRNA GENE (*E. coli* POSITION 27-680)**	
27F	AGAGTTTGATCMTGGCTCAG
680R	GAAATTCTACCCCCCTCTACAG
**TOTAL *Vibrio***	
Forward (587F)	GGCGTAAAGCGCATGCAGGT
Reverse (680R)	GAAATTCTACCCCCCTCTACAG
**MBAY Vib4**	
Forward (Vib4F)	GGGAGGAAGGTGTTGTCGTTAA
Reverse (Vib4R)	ACGGAGTTAGCCGGTGCTT
Probe (Vib4P)	[FAM]AGCGGCAGCATTTGACGTTACCCAC[TAMRA]
**MBAY Vib7**	
Forward (Vib7F)	GCAGTGAGGAAGGGTGTGTAGT
Reverse (Vib7R)	GAGTTAGCCGGTGCTTCTTCTG
Probe (Vib7P)	[FAM]ATAGCTGCATACTTTGACGTTAGCTG[TAMRA]

*The 16S rRNA gene and Total Vibrio primers are from Thompson et al. (2004a,b). The MBAY Vib4 and MBAY Vib7 primer and FAM/TAMRA labeled probe sets were developed based on sequences from the Monterey Bay 16S rRNA gene clone libraries*.

### Quantitative PCR (qPCR)

Total *Vibrio* abundances were quantified using a SYBR Green qPCR method utilizing the *Vibrio* specific primers 567F and 680R (Table 1) from Thompson et al. (2004b) to ensure a broad spectrum of *Vibrio* species were included in the analysis. DNA template (2 μ l) was added to 12.5 μ l Applied Biosystems SYBR® Green PCR Master Mix, 8.25 μ l of 5 kD filtered water, 0.25 μ l of bovine serum albumin (BSA) and 1 μ l of each primer for a total volume of 25 μ l. The cycling conditions were 50°C for 2 min, 95°C for 10 min followed by 40 cycles of 95°C for 15 s and 64°C for 1 min. Each run was followed by a dissociation step (95°C for 15 s and 60°C for 1 min 95°C for 15 s and 60°C for 15 s) to determine a melt curve for analysis of specificity.

Two qPCR assays utilizing TaqMan® chemistry were designed to target two *Vibrio* subpopulations within MB based on sequences of interest identified from the MB 16S rRNA clone library. The MBAY Vib4 group was closely associated with *Alllivibrio* sp. and the MBAY Vib7 group was associated with *Vibrio penaecida*. Primers and probes were designed with Primer Express (Table 1) (ABI, Foster City, CA) and synthesized by Sigma-Genosys (Woodlands, TX). The reactions for the sub-population assays contained 2 μ l of sample DNA, 12.5 μ l Applied Biosystems TaqMan® Universal PCR Master Mix, 8 μ l of 5 kD filtered water, 1 μ l each of the forward and reverse primer and 0.5 μ l of the probe. Samples were assessed using a TaqMan® qPCR method with cycling conditions of 50°C for 2 min, 95°C for 10 min followed by 45 cycles of 95°C for 15 s and 60°C for 1 min. The qPCR primer sets were used to analyze the full range of samples at both stations.

Both qPCR assays compared *C*_T_ values to standard curves (equivalent to 10^1^–10^8^ gene copies per reaction) derived from group specific environmental isolate clones from the MB clone library linearized with Nad1. An internal control at a concentration of 10^4^ gene copies of linearized standard assessed the runs for inhibition. If detected, DNA samples were diluted 1:10 and amplification was repeated. All qPCR runs were performed on a 7500 Applied Biosystems Real-Time PCR instrument. Final concentrations are reported as gene copies l^−1^ of seawater sampled.

### Statistical analysis

Statistical analysis was performed in JMP version 9 (SAS Institute, North Carolina). Data was natural log transformed [ln (x + 1)] to scale variables for graphical interpretation and adjust for normalcy. A Principal Components Analysis (PCA) bi-plot was utilized to visualize relationships between *Vibrio* populations and physical and biological variables and Spearman's rank analysis (ρ_s_) was used to define statistically significant relationships.

## Results

### Environmental conditions in MB

**Figure 2:**
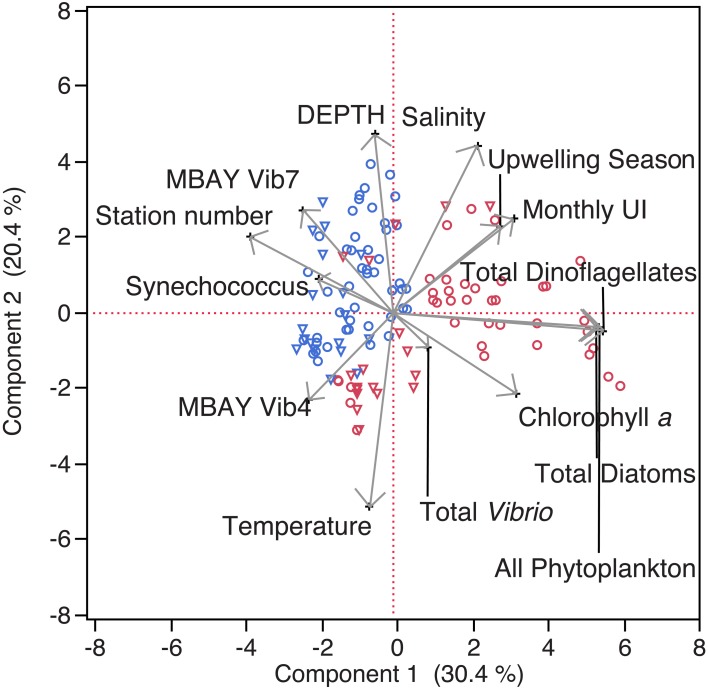
**Principal Component Analysis of physical and biological parameters**. MBAY Vib4 (**O**) MBAY Vib7 (Δ) from stations C1 (red) and M2 (blue).

Water temperatures from the sample sites ranged from 7.7 to 15.4°C (Table 2). Salinity ranged from 32.7 to 34.4 ppt and chlorophyll *a* values ranged from 0 to 18.45 μ g liter^−1^ (Table 2). The Monthly Upwelling Index (MUI) ranged from 46 to 237 m^3^ s^−1^ 100 m of coastline^−1^. The daily Upwelling Index was analyzed against surface conditions at M2 (Table 3A). Results were consistent with expected trends for upwelling events with a negative correlation to temperature (ρ_s_ = −0.47, *p* = 0.002) and a positive correlation to both nitrate (ρ_s_ = 0.22, *p* = 0.035) and chlorophyll *a* (ρ_s_ = 0.28, *p* = 0.009). Principal Component Analysis associated total diatoms, total dinoflagellates and all phytoplankton with the upwelling season and the monthly UI. *Synechococcus* was more closely associated with station M2 (Figure 2).

**Table 2:** **CTD measurement and *Vibrio* concentration ranges over the study period for all stations and depths**.

**Station**	**Depth**	**Temperature**	**Salinity**	**chlorophyll *a***	**Total *Vibrio***	**MBAY Vib4**	**MBAY Vib7**
		**(°C)**	**(ppt)**	**(μ g m^−3^)**	**(gene copies l^−1^ seawater)**		
All	All	7.7–15.4	32.7–34.4	0.00–18.45	1.80 × 10^3^–3.72 × 10^6^	nd-1.95 × 10^6^	nd-5.18 × 10^5^
C1	5	10.7–14.7	33.4–33.9	0.76–18.45	2.14 × 10^4^–3.72 × 10^6^	nd-1.95 × 10^6^	nd-1.07 × 10^4^
	10	10.5–14.3	32.7–34.0	0.88–6.61	2.20 × 10^4^–2.28 × 10^6^	nd-4.03 × 10^5^	nd-2.22 × 10^4^
	20	9.4–13.7	32.9–33.9	0.01–9.17	2.05 × 10^3^–2.83 × 10^6^	nd-1.54 × 10^6^	nd-1.69 × 10^4^
	30	8.9–13.4	33.2–34.0	0.00–2.87	5.08 × 10^3^–2.57 × 10^6^	nd-1.02 × 10^5^	nd-5.05 × 10^4^
	200	7.7–10.0	33.9–34.2	0.00–0.34	1.05 × 10^5^–1.90 × 10^6^	nd-4.83 × 10^5^	nd-3.92 × 10^4^
M2	5	10.7–15.4	33.3–34.0	0.39–8.59	3.42 × 10^3^–3.13 × 10^6^	nd-3.99 × 10^4^	nd-9.35 × 10^4^
	10	10.5–15.1	33.3–33.9	0.60–9.84	2.23 × 10^3^–1.01 × 10^6^	nd-2.95 × 10^4^	nd-5.50 × 10^4^
	20	10.5–14.6	33.2–33.7	0.40–5.18	1.80 × 10^3^–2.58 × 10^5^	nd-2.68 × 10^4^	nd-6.58 × 10^4^
	40	9.7–13.3	33.0–33.8	0.05–1.38	2.18 × 10^4^–4.77 × 10^5^	nd-5.78 × 10^4^	nd-1.09 × 10^5^
	100	8.6–10.6	33.7–34.0	0.00–0.34	7.18 × 10^3^–7.13 × 10^5^	nd-3.02 × 10^4^	nd-5.18 × 10^5^
	200	7.9–9.4	34.0–34.4	0.00–0.17	2.29 × 10^4^–6.92 × 10^5^	nd-1.63 × 10^4^	nd-1.07 × 10^5^

*nd, none detected*.

**Table 3:** **Spearman's rho (ρ_s_) statistical analysis of physical and biological parameters**.

**(A) UPWELLING PARAMETERS**			
	**Temperature**	**Chlorophyll *a***	**Nitrate**
UI	**−0.47**	**0.28**	**0.22**
Nitrate	**−0.53**	**0.15**	
Chlorophyll *a*	**−0.20**		
**(B) CTD AND ENVIRONMENTAL PARAMETERS**			
	**Total *Vibrio***	**MBAY Vib4**	**MBAY Vib7**
Station number	**−0.32**	**−0.16**	**0.53**
Depth	0.15	0.00	**0.41**
Temperature	**−**0.03	**0.32**	**−0.30**
Salinity	0.05	**−0.22**	0.12
Chlorophyll *a*	**−**0.10	**−0.16**	**−0.47**
Sample day UI	0.02	**−0.02**	**−0.18**
Monthly UI	**−0.26**	**−0.61**	**−0.23**
Davidson season	0.06	0.19	**0.15**
Oceanic season	**0.31**	**0.41**	0.03
Upwelling season	**−0.21**	**−0.51**	**−**0.10
Year of study	**−**0.14	**−0.35**	**−**0.09
MBAY Vib7	0.01	0.01	
MBAY Vib4	**0.55**		
**(C) SURFACE PARAMETERS**			
	**Total *Vibrio***	**MBAY Vib4**	**MBAY Vib7**
All phytoplankton	0.18	**−0.44**	**−0.38**
Total diatoms	0.10	**−0.53**	**−0.43**
Total dinoflagellates	0.17	**−**0.17	**−0.43**
*Synechococcus*	0.02	**0.20**	**0.41**
Surface temp.	0.21	**0.45**	0.07
Surface Chlorophyll *a*	**−**0.21	**−0.30**	**−0.29**
Surface nitrate	**−**0.20	**−0.39**	0.00

*Significant parameters (p ≤ 0.05) are in bold type. UI, upwelling index. (A) Upwellling parameters verified against surface data from the M2 mooring station. (B) CTD data and environmental parameters. (C) Surface phytoplankton and M2 mooring station data*.

### 16S rRNA gene phylogenetic analysis

**Figure 3:**
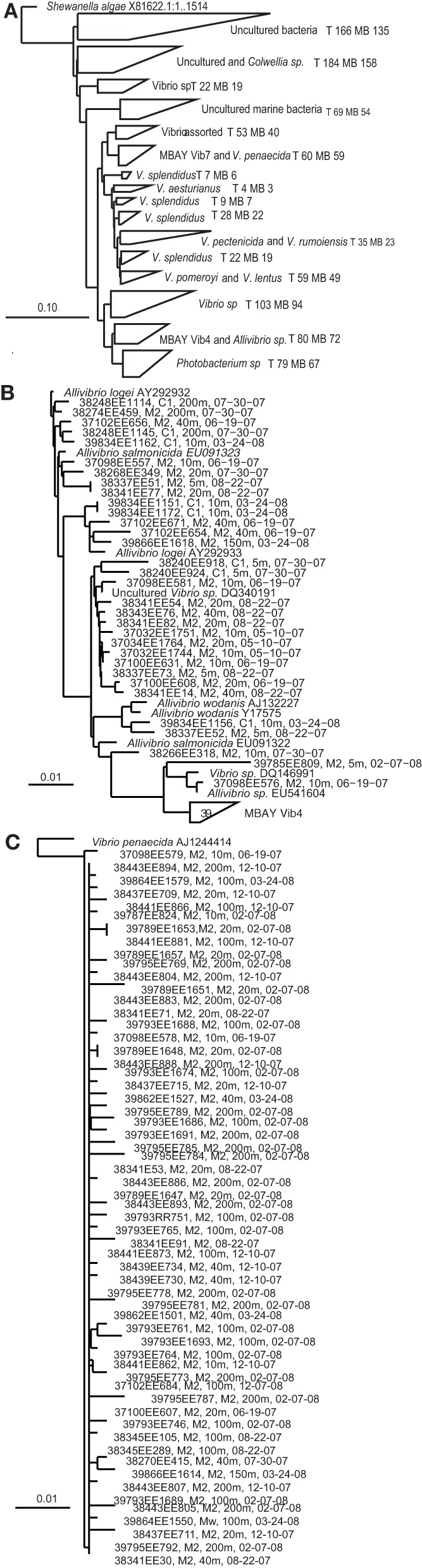
**Neighbor-joining phylogenetic trees of *Vibro* sequences from Monterey Bay at stations C1 and M2**. Tree constructed in ARB from partial 16S rRNA sequences (*E. coli* 27-680 bp) using ARB neighbor-joining distance matrix method with a Jukes-Cantor correction. T, total number of sequences in clade, MB, MB sequences in clade. Sample number, station, depth, and date are listed for Monterey Bay sequences. **(A)** Major groups of environmental *Vibrio* sequences recovered from the MB. *Shewanella algae* included as the tree root. **(B)** *Allivibirio* and MBAY Vib4 group sequences, and **(C)** *Vibrio penaecida* and MBAY Vib7 sequences. Scales represent 10% or 0.1% nucleotide substitutions.

Multiple distinct 16S rRNA-based phylotypes were recovered from the MB clone libraries (Figure 3). The primer sets utilized to develop the MB clone libraries targeted *Vibrio* sp. strains but 67 sequences within the library clustered with the closely related *Photobacterium* sp. There were also a significant number of sequences that clustered with unidentified *Vibrio* strains and uncultured marine bacteria. *Vibrio splendidus* was defined in several distinct clades within the phlogenetic tree. Other MB sequences clustered with *V. pomeroyi* and *V. lentus*; *V. pectenicida* and *V. rumoiensis*; and *V. aesturianus*. One clade included sequences that clustered with *V. agarivorans, V. hispanicus, V. haliticoli, V. ezurae, V. fortis, V. proteolyticus, V. sinaloensis, V. nigripulchritudo, V. harveyi, V. natriegens*, and *V. alginolyticus* (Assorted Vibrio in Figure 3). Quantitative PCR (qPCR) assays were developed for two specific phylotypes recovered from the MB clone libraries. The MBAY Vib4 group (Figure 3B) clustered with *Allivibrio* sp., which includes *A. salmonicida*, *A. fischeri*, *A. wodanis*, and *A. logei*. MBAY Vib4 included sequences obtained from samples collected from both stations C1 and M2. Within the clone library the MBAY Vib7 group included sequences that were only obtained at station M2 and clustered with *Vibrio penaecida* (Figure 3C).

Rarefaction curves were determined based on the neighbor-joining distance matrix of sequences within the MB clone libraries. Of the 827 sequences included in the total sequences analysis, 158 OTUs were defined at 97% identity, 103 OTUs at 95% identity, 61 OTUs at 93% identity, and 30 OTUs at 90% identity. Only at 93 and 90% identity did the rarefaction curves begin to reach an asymptote. Analysis revealed that there was no significant difference in species richness between station C1 and M2 but that both sampling efforts did not attain full coverage to estimate total population OTUs. Rarefaction analysis of the 327 sequences from station C1 defined 97 OTUs at 97% identity, 69 OTUs at 95% identity, 45 OTUs at 93% identity, and 23 OTUs at 90% identity. Rarefaction analysis of the 500 sequences from station M2 defined 98 OTUs at 97% identity, 66 OTUs at 95% identity, 41 OTUs at 93% identity, and 21 OTUs at 90% identity.

### Quantification of *Vibrio* population abundances

**Figure 4:**
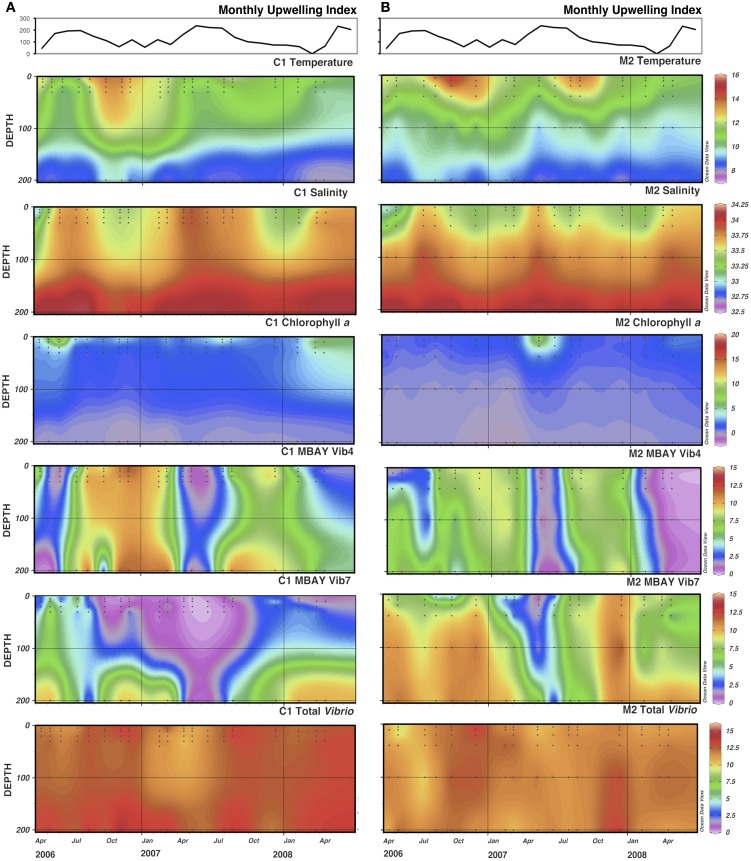
**Monthly Upwelling Index (m^3^ s-^1^ 100 m of coastline-^1^), temperature (°C), salinity (ppt), chlorophyll *a* (μg m^−3^) and total *Vibrio*, MBAY Vib4 and MBAY Vib7 concentrations (natural log of gene copies liter^−1^) for stations C1 (A) and M2 (B)**. Black dots represent discrete samples within the CTD cast.

There were observed differences in the *Vibrio* populations at both of the stations, with the MBAY Vib4 group having higher peak abundances at station C1 (Figure 4). The MBAY Vib4 group ranged in concentration from undetectable levels at all depths and stations up to 1.95 × 10^6^ gene copies liter^−1^ seawater at 5 m at station C1 (Table 2). MBAY Vib4 was positively correlated to temperature (ρ_s_ = 0.32, *p* = 0.002), oceanic season (ρ_s_ = 0.41, *p* = 0.002), *Synechococcus* (ρ_s_ = 0.20, *p* = 0.02), and total *Vibrio* abundance (ρ_s_ = 0.55, *p* < 0.001) (Table 3). This group was negatively correlated to salinity (ρ_s_ = −0.22, *p* = 0.02), chlorophyll *a* (ρ_s_ = −0.16, *p* = 0.035), monthly UI (ρ_s_ = −0.61, *p* < 0.001), surface phytoplankton (ρ_s_ = −0.44, *p* < 0.001), and total diatoms (ρ_s_ = −0.55, *p* < 0.001) (Table 3).

The MBAY Vib7 population was not as abundant as MBAY Vib4 (Figure 4), with concentrations ranging from undetectable to 5.18 × 10^5^ gene copies liter^−1^ seawater (Table 2). MBAY Vib7 was positively correlated with depth (ρ_s_ = 41, *p* < 0.001) (Table 3), with the highest detected concentration at 100 m at station M2 (Table 2). MBAY Vib7 populations were positively associated with station M2 (ρ_s_ = 0.53, *p* < 0.001), surface *Synechococcus* populations (ρ_s_ = 0.41, *p* < 0.001), and the Davidson season (ρ_s_ = 0.15, *p* = 0.0486) (Table 3). Temperature (ρ_s_ = −0.30, *p* = 0.0005), chlorophyll *a* (ρ_s_ = −0.47, *p* < 0.001) and monthly UI (ρ_s_ = −0.23, *p* = 0.012) were all negatively associated with MBAY Vib7 (Table 3). MBAY Vib7 was identified in samples from both station C1 and M2 but at lower concentrations and at deeper depths at station C1.

Total *Vibrio* populations showed some variability in abundance but were consistently present throughout the study at all depths and stations (Figure 4). Total *Vibrio* population abundances ranged from 1.80 × 10^3^ to 3.72 × 10^6^ gene copies liter^−1^ seawater (Table 2). The abundance of total *Vibrio* populations was positively correlated to MBAY Vib4 (ρ_s_ = 0.55, *p* < 0.001), and the oceanic season (ρ_s_ = 0.31, *p* < 0.001) and negatively correlated to station number (ρ_s_ = −0.32, *p* < 0.001) the monthly UI (ρ_s_ = −0.26, *p* = 0.0039) and upwelling season (ρ_s_ = −0.21, *p* = 0.0067) (Table 3). No other parameters examined in this study showed significant correlation with the abundance of the total *Vibrio* population.

## Discussion

Genetic diversity within *Vibrio* populations can be up to 7% when examining the entire 16S rRNA gene (Dorsch et al., 1992; Kitatsukamoto et al., 1993). This variability is mostly located within 35 defined regions, 28 of which are located between the primers used to construct the clone library for this study (Wiik et al., 1995; Jensen et al., 2009). It is thus not surprising to have identified a significant number of OTUs and to observe deep branching within the phylogenetic tree of the MB clone library. Intraspecies sequence variability can also be high within *Vibrio* species (Jensen et al., 2009) and the identification of *Vibrio splendidus* within different branches of the tree is supported by observations of up to 2% difference within the 16S rRNA gene of this species (Jensen et al., 2009; Le Roux et al., 2009). Defining relationships within *Vibrio* populations is even more complex as their genomes contain multiple copies of the rRNA operon (Heidelberg et al., 2000). Thirteen copies have been identified in *V. natriegens* and 12 copies in both *Allivibrio fischeri* and *A. salmonicida* (Lee et al., 2009). Intragenomic heterogeneity within these copies may be high, as seen in *V. parahemolyticus* (Harth et al., 2007), or non-existent as is noted in other *Vibrio* species (Coenye and Vandamme, 2003). Since this study was not designed to differentiate between heterogeneous intragenomic copies and individuals, our results may overestimate diversity within the MB clone library. With that being said, sequences did cluster with over 20 known *Vibrio* species suggesting that MB does in fact contain diverse *Vibrio* populations.

Multiple copies of the rRNA operon may also affect the qPCR estimates of abundance presented in this study, but is not expected to significantly change the interpretations of our findings. Even an estimate of 20 gene copies per cell results in estimates of abundances that fall within the range of concentrations observed in other coastal waters (Thompson et al., 2004a), and are higher than observations from samples in similar temperature ranges (Randa et al., 2004; Eiler et al., 2006). Temperature is often cited as a significant driver in *Vibrio* population abundance (Heidelberg et al., 2002; Maeda et al., 2003; Eiler et al., 2006, 2007; Hsieh et al., 2008), and while temperature variability was correlated to the two subpopulations examined in this study, it was the upwelling index (MBAY Vib4) and depth and station (MBAY Vib7) that were more significant factors in defining subpopulation abundance.

MBAY Vib4 and MBAY Vib7 are separate subpopulations of the *Vibrio* community with distinct niches in the MB. The MBAY Vib4 is defined by close association with *Allivibrio* sp. and represents an oceanic season subpopulation. This season is characterized by a lack of upwelling, lower sea surface salinities, warmer sea surface temperatures and waters that are influenced by wind relaxation events and the slow flowing California Current (Rosenfeld et al., 1994). MBAY Vib7 represents a deeper water subpopulation of *Vibrio* sp. with a greater association with the offshore waters at station M2. This station is influenced by upwelled waters mixed with California Current waters, the California Undercurrent and the Davidson Current-a northward flowing current that develops in winter months (Tisch et al., 1992; Breaker and Broenkow, 1994; Rosenfeld et al., 1994). The California Undercurrent usually flows below 100 m along the California shelf but often nears the surface during the Davidson season (Tisch et al., 1992; Pierce et al., 2000; Tseng et al., 2005). The largest hydrographic changes around MB occur during transitions between upwelling regimes (i.e., during oceanic phases) and in winter when the horizontal and vertical thermal gradients are reduced due to the northward flow of the Davidson Current (Bac et al., 2003; Storlazzi et al., 2003; Warn-Varnas et al., 2007). Dynamics of the MB *Vibrio* populations examined in this study seem to reflect these hydrographic processes, and may be highly influenced by them-MBAY Vib4 during the oceanic phases and MBAY Vib7 during the winter phases.

In 2006, the MB had experienced 4 years of anomalous oceanographic conditions. Delayed and unusually shallow upwelling affected the food web in MB up through higher trophic levels (Goericke et al., 2007). Changes included a shift in dominant toxin-producing algal species from diatoms to dinoflagellates, poor recruitment of krill and low zooplankton biomass as well as seabird reproductive failure (Peterson et al., 2006; Goericke et al., 2007; Jester et al., 2009). MBAY Vib4 was positively correlated to this year possibly due to delayed upwelling, reduced chlorophyll *a* concentrations and warmer surface temperatures (Table 3). Increased abundances of *Vibrio* populations could also have been influenced by decreased competition from phytoplankton for resources or reduced top down control from zooplankton. Following the 2002–2006 lull events, unusually strong upwelling was observed in 2007 (Kaplan et al., 2009), and in 2008 a strong development of upwelling was observed at the beginning of the season (Figure 3). MBAY Vib4 populations showed significantly reduced abundance during those upwelling seasons. MBAY Vib7 and the total *Vibrio* populations did not seem to be strongly affected by these events.

Overall, the *Vibrio* populations examined in MB seemed to be influenced by larger scale upwelling events and shifts in currents and oceanographic seasons rather than individual environmental factors. In the future, more extensive analysis of nutrient availability and oceanographic parameters (i.e., flow velocities and wind patterns) may provide better insight into how upwelling characteristics and water mass flows play a role in *Vibrio* population dynamics. Since *Vibrio* populations might have significant influence over nutrient availability through recycling chitin and other bound nutrient resources, understanding the role of these bacterial populations in both surface and deeper waters can better our understanding of the productivity potential within upwelling regions.

### Conflict of interest statement

The authors declare that the research was conducted in the absence of any commercial or financial relationships that could be construed as a potential conflict of interest.
